# Heuristics Identified in Health Data–Sharing Preferences of Patients With Cancer: Qualitative Focus Group Study

**DOI:** 10.2196/63155

**Published:** 2024-12-17

**Authors:** Anna Hermansen, Samantha Pollard, Kimberlyn McGrail, Nick Bansback, Dean A Regier

**Affiliations:** 1 BC Cancer Research Institute Vancouver, BC Canada; 2 School of Population and Public Health University of British Columbia Vancouver, BC Canada

**Keywords:** heuristics, health data sharing, cancer patients, decision-making, real-world data, altruism, trust, control, data sharing, focus group, precision medicine, clinical data, exploratory study, qualitative, Canada, thematic analysis, informed consent, patient education, information technology, healthcare, medical informatics

## Abstract

**Background:**

Evaluating precision oncology outcomes requires access to real-world and clinical trial data. Access is based on consent, and consent is based on patients’ informed preferences when deciding to share their data. Decision-making is often modeled using utility theory, but a complex decision context calls for a consideration of how heuristic, intuitive thought processes interact with rational utility maximization. Data-sharing decision-making has been studied using heuristic theory, but almost no heuristic research exists in the health data context. This study explores this evidence gap, applying a qualitative approach to probe for evidence of heuristic mechanisms behind the health data-sharing preferences of those who have experienced cancer. Exploring qualitative decision-making reveals the types of heuristics used and how they are related to the process of decision-making to better understand whether consent mechanisms should consider nonrational processes to better serve patient decision-making.

**Objective:**

This study aimed to explore how patients with cancer use heuristics when deciding whether to share their data for research.

**Methods:**

The researchers conducted a focus group study of Canadians who have experienced cancer. We recruited participants through an online advertisement, screening individuals based on their ability to increase demographic diversity in the sample. We reviewed the literature on data-sharing platforms to develop a semistructured topic guide on concerns about data sharing, incentives to share, and consent and control. Focus group facilitators led the open-ended discussions about data-sharing preferences that revealed underlying heuristics. Two qualitative analysts coded transcripts using a heuristic framework developed from a review of the literature. Transcripts were analyzed for heuristic instances which were grouped according to sociocultural categories. Using thematic analysis, the analysts generated reflexive themes through norming sessions and consultations.

**Results:**

A total of 3 focus groups were held with 19 participants in total. The analysis identified 12 heuristics underlying intentions to share data. From the thematic analysis, we identified how the heuristics of social norms and community building were expressed through altruism; the recognition, reputation, and authority heuristics led to (dis)trust in certain institutions; the need for security prompted the illusion of control and transparency heuristics; and the availability and affect heuristics influenced attitudes around risk and benefit. These thematic relationships all had impacts on the participants’ intentions to share their health data.

**Conclusions:**

The findings provide a novel qualitative understanding of how health data–sharing decisions and preferences may be based on heuristic processing. As patients consider the extent of risks and benefits, heuristic processes influence their assessment of anticipated outcomes, which may not result in rational, truly informed consent. This study shows how considering heuristic processing when designing current consent mechanisms opens up the opportunity for more meaningful and realistic interactions with the complex decision-making context.

## Introduction

IT in health care has generated a wide range of digital patient data collected during various health-related activities, including medical interactions, diagnoses, quality of life, treatments, drug adherence, and reimbursement [1-3]. Accessing real-world data allows for a detailed profile of a patient to better understand and target health issues and build evidence to understand the clinical and cost-effectiveness of personalized health technologies [4]. Access to clinical and routinely collected real-world data hinges on patient consent and legislation. As health institutions look toward data-sharing solutions that can support evidence generation for personalized care, they must consider how to best incorporate patient concerns and expectations to reach meaningful consent [5-9].

Understanding an individual’s process of providing consent involves modeling attitudes, context, and risks and benefits involved in the decision [10,11]. Decision-making modeling is typically grounded in utility theory, where rational actors deliberate on the outcomes of possible alternatives and make a decision that maximizes their utility, based on well-defined and consistent preferences and using unlimited cognitive processing power [12-15]. Simon [14,16] (1957, 1979) provided an alternative framework, arguing that in a complex and uncertain decision context, individuals hit a cognitive limit to their rational processing capabilities and instead satisfice their utility. The individual’s choice is based on the context-specific value placed on the potential outcomes rather than a rational assessment of their utility function [17-19].

**Figure 1:**
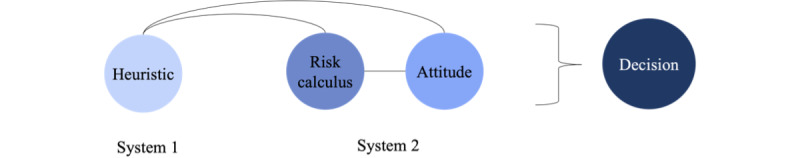
Conceptual model of heuristic decision-making.

Kahneman and Tversky [15,18] (1974) explored satisficing through heuristic theory and defined heuristics as the different rules of thumb used by individuals to substitute rational processing for quicker judgment calls. Kahneman [20] (2003) elaborated on the decision-making process as a dual-system model where system 1 rational (controlled, slow) and system 2 heuristic (intuitive, automatic) processes react during complex decision-making. The authors used this dual-system theory to develop Figure 1, which represents decision-making as an interaction among heuristics, attitudes, and the risk-benefit calculus, all of which are defined in Table 1 [21,22]. In this study, we take the position that the decision to share data is a complex process that involves the interaction of both heuristic and rational processes. In this map, the system 1 heuristic process interacts with the system 2 attitudes and risk-benefit calculus to form the decision to share data. The relationships between systems 1 and 2 are not reducible to a simple cause and effect; both systems nondirectionally influence each other to lead to a decision.

**Table 1:** Definitions of variables in the conceptual model of heuristic decision-making.

Variable	Definition
Heuristics	Judgment calls replacing rational processing with intuitive responses
Attitudes	The way individuals feel about a certain behavior; includes feelings, experiences, awareness, situational cues, and dispositions
Risk calculus	Rational deliberation between the risk and benefit involved in a decision
System 1	Intuitive, involuntary processing of concepts to generate impressions
System 2	Effortful, explicit processing of concepts to generate deliberate reasoning
Decision	Interaction between systems 1 and 2

Published research has found the presence of various heuristic processes in data-sharing decision-making [10,23-26]. However, very few have studied heuristics specifically used in health data sharing [7], meaning that the impact of heuristics on a patient’s consent to share health data is not well understood. This study addresses this evidence gap by asking what heuristics, if any, are used when deciding whether to share personal health data for research purposes and how they impact the outcome of a stated decision. We are investigating the presence of heuristic processes to understand whether patients may be using a decision process similar to Figure 1. Capturing the use of heuristic processing in the health data–sharing context helps illuminate whether consent processes can better serve patient decision-making by considering the factors that influence patients beyond their rational, utility-maximizing processes. These findings can support the development of consent mechanisms that represent the reality of this complex and context-dependent decision environment [5].

## Methods

### Overview

We conducted a qualitative focus group study with patients with cancer and survivors in Canada as part of the Canadian Network for Learning Healthcare Systems and Cost Effective ‘Omics Innovation (CLEO) project, an initiative evaluating 6 precision oncology programs in Canada to inform the design of a learning health care system for cancer research and care [27]. Patients with cancer were the chosen population for this focus group study because of their relevance to the CLEO project and convenience sampling for the study authors who worked at a cancer research institute. Focus groups were chosen because they are effective for collecting data on socially construed beliefs, for exploring concepts that are not easily measurable, and for revealing underlying thought processes [24,28,29]. As compared with interviews, their social setting allows for discussion that is influenced by others’ perspectives [30]. This discussion style can provide mental cues for participants, triggering memories, opinions, or feelings that represent heuristic processing.

### Ethical Considerations

The protocol for data collection and analysis received ethics approval from the University of British Columbia BC Cancer Research Ethics Board (H20-00861). Participation in the focus groups was entirely voluntary. Consent for participation was collected through a consent form that described the goals of the study, the research team, the format of the focus group, expectations of participants, compensation, how confidentiality will be protected, and who to contact for more information. Consent forms were delivered by email to each participant before the focus group. The participants signed their forms as a PDF or through REDCap (Research Electronic Data Capture, Vanderbilt University), and only received the Zoom (Zoom Video Communications) link once they had completed the consent form. A copy of the consent form can be found in Multimedia Appendix 1. The participants received financial compensation of CAD $100 (US $70) after participating.

This study presented minimal risk to the participants. All demographic data collected from the participants through REDCap was stored in the REDCap platform and a locked folder on secure BC Cancer servers. We removed participant names from the transcripts and replaced them with anonymous identifiers. We stored the de-identified transcripts in a locked folder on BC Cancer servers.

### Study Procedure

Individuals with a current or previous cancer diagnosis were recruited through a purposive sampling technique across provinces in Canada [30]. Participants were deliberately sampled to maximize diversity and not representativeness of the Canadian population. We recruited a diverse sample of cancer type, age, gender, and racial or cultural groups in order to hear from a wide range of perspectives. The participants were recruited from an advertisement posted on various provincial patient organizations (REACHBC, AbSPORU, and Cancer Care Ontario) and Kijiji, Craigslist, and Facebook patient groups. Oncologists at BC Cancer also supported purposive recruitment for racial and cultural diversity and cancer type. Those who expressed interest in participating were sent a screening survey that collected gender, age, racial or cultural group identity, cancer type, and general availability (a copy of the survey is mentioned in Multimedia Appendix 2). As screened candidates entered our selection process, we built a list based on those who provided variation in these demographic categories we collected, and we excluded those who did not provide demographic diversity compared with those previously selected. Those who were selected were invited to participate in a single focus group. They were sent the consent form and an introductory video on precision oncology and data-sharing platforms.

Focus groups were 90-minute sessions held and recorded over Zoom, facilitated by 2 female researchers at their workplace (SP and AH). At the time of the study, SP was a senior methodologist at BC Cancer with a PhD in epidemiology, ethics, and mixed-methods research. AH was a graduate student in public health with a bachelor’s degree in political science with no prior experience conducting focus groups. No relationship was established with the participants prior to the focus groups beyond the communication required to set up their participation.

The facilitators used a semistructured question guide informed by a literature review [31] to guide the discussions on preferences for the data-sharing process (Multimedia Appendix 3). The literature review identified key topic areas and evidence gaps in the study of preferences for secured data-sharing platforms. These included motivators for and concerns about sharing data, incentives to allow access, types of institutions that can access the data, and consent and control features. The facilitators used these topics to develop and prioritize questions for the focus groups. Two researchers (AH and DAR) also developed questions to probe for heuristic processing, although the open-ended nature of the conversations was enough to naturally reveal heuristic processing. After the focus groups, the participants completed a demographic survey and were sent a CAD $100 (US $70) honorarium. Transcripts were not returned to participants.

### Data Analysis

Data collection and analysis happened concurrently to achieve constant comparison [32] and followed a thematic analysis approach. Thematic analysis is a qualitative practice used for identifying and interpreting patterns across the data on participants’ experiences, views, perspectives, and behaviors [33]. We chose this method because of the flexibility it offers in terms of theoretical approach and data analysis, allowing for a theoretical (deductive) application of a previously established theory to a novel research area [34]. Because this area is novel, we chose to extend our methods beyond cataloging or describing the results as topic summaries, and into the interpretation of these results, generating themes by reflexively identifying underlying motivators for the heuristic processing identified [35].

The research team worked to maintain a degree of transferability, dependability, and reflexivity in the data collection, analysis, and reporting [36]. Using the COREQ (Consolidated Criteria for Reporting Qualitative Research) standards, the explanation of the research setting and definition of heuristics increases the transferability of the study, and the documentation of our data collection and analysis processes encourages dependability of the findings [37]. This work also incorporated reflexive activities including team debriefing to review field notes after each focus group, weekly norming sessions, and working group consultations at major points along our study process [30].

We conducted a deductive analysis guided by a heuristic framework we developed from a review of literature on data-sharing heuristics. After focus group recordings were de-identified, transcribed, and checked for quality, 2 researchers (AH and SP) independently open-coded the transcripts, tagging the data for instances of the heuristic framework. Throughout the coding process, we refined and grouped heuristics to establish the final codebook. Statements could be coded with more than one heuristic. AH and SP conducted the coding process using QSR NVivo Software [38]. We completed data collection after reaching saturation, which we defined as the moment when analysis of new transcripts led to the same heuristic findings, with the same or similar impacts on the decision to share [39].

Once coding was complete, we looked for phrases in relevant passages of the transcript to identify the heuristics’ corresponding attitudes and intentions to share, following the decision-making model (Figure 1). Heuristic codes were grouped into categories that were inductively created by identifying what causes the heuristic to be triggered and then defining larger sociocultural spheres of influence related to these causes. Resulting themes were derived from the data, representing each of these categories’ heuristic influences on participants’ intentions to share their data. The participants did not provide feedback on the heuristic findings.

## Results

In total, 3 researchers (AH, SP, and DAR) conducted 3 Zoom-based focus groups from January to April 2022. Overall, 19 participants took part in the focus groups, with 5 to 8 participants per group. One candidate completed the consent form and received the Zoom link but did not attend the focus group. A total of 4 candidates confirmed their availability for the focus group but ended communication before completing the consent form. Table 2 describes participant demographic characteristics.

We coded 12 heuristics in the transcripts following the codebook (Table 3). Heuristics each have their own definitions but overlap in how and why they are triggered and what outcomes they produce. The differences between some of these heuristics are nuanced and the researchers relied on definitions from literature to maintain distinctions between them. We generated 4 themes linked to 9 heuristics. The other 3 heuristics identified were minor, nonsaturated findings (gatekeeping, novelty, and representativeness), and were not included in the thematic analysis.

**Table 2:** Self-reported participant demographics.

Demographics (N=19)			Values
**Gender, n (%)**			
	Men	11 (58)	
	Women	8 (42)	
Age (years), median (range)			63.5 (22-85)
**Canadian province, n (%)**			
	British Columbia	8 (42)	
	Ontario	7 (37)	
	Alberta	3 (16)	
	Manitoba	1 (5)	
**Racial or cultural group, n (%)**			
	White	12 (79)	
	East Asian	4 (21)	
	Southeast Asian	2 (11)	
	Missing	1 (5)	
**Experience with cancer, n (%)**			
	Previous diagnosis	10 (53)	
	Current diagnosis, undergoing tx^a^	6 (32)	
	Current diagnosis, not undergoing tx	2 (10)	
	Missing	1 (5)	
**Cancer type^b^**			
	Breast	6 (33)	
	Prostate	3 (17)	
	Lung	3 (17)	
	Melanoma	2 (11)	
	Bone	1 (5)	
	Thyroid	1 (5)	
	Gastric	1 (5)	
	Uterine	1 (5)	
	Cervix	1 (5)	
	Endometrial	1 (5)	
	Duodenum	1 (5)	
	CLL^c^	1 (5)	
**Education level, n (%)**			
	Bachelor’s or above	11 (58)	
	Diploma	4 (21)	
	University below bachelor’s level	3 (16)	
	High school	1 (5)	

^a^tx: treatment.

^b^Some participants reported more than one cancer type.

^c^CLL: chronic lymphocytic leukemia.

**Table 3:** Heuristic codebook.

Category and code^a^		Definition	Example from data
**The personal**			
	Affect	Emotional response dictates assessment; objects and events are tagged with emotion and stored in a cognitive “affective pool” that is consulted when making a decision [40]	*That gives me a warm fuzzy feeling that it’s very secure.* [P10]
	Availability	The ease of recalling something from memory, whether a frequent occurrence or a prominent event (eg, personal experience or news story) [15]	*[W]e hear it in the news all the time with hacking*. [P09]
**The social**			
	Social norms	How others’ behavior impacts the decision, or how external peer influences impact the decision [41]	*I’ve seen this in other, a lot of other people that are not going ahead with [genetic] testing.* [P04]
	Community building	Being part of a community influences individuals to trust and share information with each other, and to contribute to the common good [25]	*We have to be together trying to help each other to survive the best we can*. [P07]
**The institutional**			
	Recognition	Ascribing value to and holding an inherent attitude toward an entity that is recognized or familiar [42,43]	*The only time I care [where my information is used] is when it’s being used for profit, for like a pharmaceutical company*. [P11]
	Representativeness	Passing judgment on something because of simple or salient cues that remind the individual of something else [44]	*This whole selling and stuff, that kind of scares me and that’s sort of something like the US does*. [P04]
	Reputation	Holding an inherent attitude toward an entity due to its prominence and reputation [29]	*We are dealing with medical professionals who are highly respectable and respectful* [P16]
	Authority	Holding an inherent attitude toward something because of its authority over the subject [45,46]	*We opt in or we opt out, but then the legalese covers us for all the stuff that we don’t know as patients.* [P10]
**The informational**			
	Illusion of control	Believing that having personal controls over the data-sharing process will reduce the chance of risks involved, such as a data breach [47]	*I think informed consent would still be important just so you feel you have some control*. [P19]
	Transparency	The degree to which data-sharing practices are known to the individual increases their sense of control and fairness [25,48]	*I don’t want to say I’m 100 percent in favor of sharing data, because I don’t necessarily know where it is going to be stored*. [P05]
**The technological**			
	Novelty	Encounters with new technology, good or bad [24,28]	*in two years I guess you probably have to move [the data] to something new, a new technology, right?* [P04]
	Gatekeeping	Being confident in and trusting a system that has many layers of access [24]	*a central committee or group that is a gatekeeper to what is going to be accessed or what needs to be inputted and not.* [P18]

^a^Codes are used to identify and group raw data into categories of analysis. The codes used in this study were all previously identified heuristics, which are defined as cognitive processes that substitute the rational calculation of probabilities for intuitive judgments of the decision context. We organized these heuristic codes in categories of what influences or is influenced by the heuristic.

### The Social: Altruism as a Social Rule of Data Sharing

The first theme was the motivation to act altruistically when deciding to share data. The community building heuristic was found throughout the transcripts in participants’ intention to benefit others. This heuristic captured the attitude of collectivity that participants felt toward others experiencing cancer, wanting to contribute to a “common good” [P07], “mankind” [P17], and the “big world” [P02]. Participant 7 explained, “everybody has their own rights. But we have to be together trying to help each other to survive the best we can.”

Some participants acknowledged the benefit they had received from the contributions that had come before them and wanted to share to “make somebody’s procedure better through my experience just as others have gone before me” [P04]. The community-building heuristic engendered strong intentions to share data for the benefit of others.

The social norms heuristic was also identified in instances where a decision not to share data was criticized, implying a social rule to be altruistic. For some participants, this rule was expressed as a disbelief at individuals who are unwilling to share their data: “I can’t see how anyone would put up a fight or why they would not want it shared” [P02]. Participant 17 similarly implied that the social benefit generated by sharing data should motivate everyone: “Isn’t most of that data for the betterment of mankind?... I was just struggling to even get my head around why we wouldn’t do this.”

At times, this sentiment was found in direct contrast to discussions of risk. One participant downplayed the financial risk of being denied insurance: “human life is way more important than a mortgage, I’m sorry. We have to be part of a common good, not be selfish” [P07]. Altruism was also identified in the discussion on providing monetary incentives for sharing data, where some participants questioned why some would want compensation, and that it seemed like a “totally opposite kind of attitude” [P12]. Through their vocal belief in these behavior patterns, participants relied on the social norms heuristic in their decision processes.

### The Institutional: Trust as a Measure of Legitimacy

The second theme centered on instances where participants reacted to the type of entity accessing their data. The recognition heuristic was primarily used in reaction to for-profit companies accessing health data, expressing distrust and privacy concerns. Without prompting, the participants expressed negative associations with pharmaceutical and insurance companies, such as Participant 13 stating that insurance access “immediately turns [them] off.” Participant 18 shared,

I don’t think I have a problem with accessing all my data. But the question is who gets to access it, and then would I be able to opt out on certain [access] like a pharmaceutical company or an insurance company.

Some participants assumed for-profit to not have as high an ethical standard as other types of data requestors (such as academic researchers), tying this perception to the company’s morality, and whether their activity could be considered altruistic [P19]. This came out at times as a contrast between commercial and research activity, with one participant expressing that

…the only time I care [where my information is used] is when it’s being used for profit, for like a pharmaceutical company, that’s where I’m more hesitant … but if it’s just for research purposes, I’m happy.

P11

We coded trust in nonprofit and public researchers, as well as medical professionals, as the authority heuristic. During a conversation about managing data-sharing requests, Participant 18 recognized, “I don’t have the medical expertise in a lot of this,” and suggested, “I’d rather have a primary person or oncologist feeding me the information that I can trust a little bit more.” This demonstrated a reliance on their health care providers when considering whether to share data. Trust in researchers was also identified in instances of the reputation heuristic, where participants connected legitimacy and trust to the prestigiousness of public or nonprofit researchers and medical professionals. For example, one participant commented that

…we’re not dealing with gambling sites or anything disreputable. We are dealing with medical professionals who are highly respectable.

P16

Rather than examining the true risk of sharing with these types of entities, they relied on the recognition, reputation, and authority heuristics to evaluate their trust in them, which ultimately dictated their intentions to share.

### The Informational: Gaining Power and Security Through Control

The third theme was the need for control over the data-sharing process. We coded the illusion of control heuristic if the participant believed that having personal controls over the data-sharing process reduced the probability of risks. Some participants expressed an interest in having controls in place to decide who gets access to what data and under what conditions to prevent a data leak. There appeared to be support for informed, detailed, and dynamic consent, which was valued by one participant to “give people some sense of control over what they want to share and what they don't want to share” [P19]. Other participants perceived a lack of control, with the understanding that “the second [your data] leaves you you’re no longer in control of that information” [P18]. In the face of this lack of control, these oversight features were important to our participants and gave them a sense of power over the data-sharing process, despite this kind of control not necessarily minimizing the risk.

This desire for controls was often connected to participants’ need to know more about the data request before allowing access, which we identified as the transparency heuristic. Some wanted to know about the storage of data:

I don’t want to say I’m 100 per cent in favor of sharing data, because I don’t necessarily know where it is going to be stored.

P05

Other participants wanted details on why and how the data was being used:

I need to understand the reasoning behind why on each and every aspect or piece of data. Then I would be all in.

P13

Being “all in” indicates a preference based on a heuristic response to the opacity of the data-sharing process, rather than on a deliberation of risks and benefits once receiving that detailed information. Whether in the form of information on or involvement in the data-sharing process, the perception of control was an important component of our participants’ decision-making processes to mitigate risk.

### The Personal: Framing Risk and Benefit Through Personal Experiences

When explaining their decision-making processes, participants shared anecdotal experiences, feelings, and externally influenced beliefs tied to data sharing. Found across all focus groups was a reliance on the availability heuristic to evaluate the risk of cyberattacks. In these discussions, the participants recalled news stories that negatively impacted their intentions to share data:

Even just reading the paper today, you know that people are always trying to get at your data.

P05

This also included anecdotes that participants recalled from memory:

[U]nfortunately we just can’t trust everybody today because look at what we see in the world, right? … if you remember that – a few years ago where somebody left important data on a bus, of all things, that was in Vancouver, you know.

P04

These stories led the participants to believe that data breach was an inevitability, with one expressing, “it seems there is always something somewhere that goes wrong” [P04]. The inevitability and frequency of a breach were a substantial consideration for our participants when discussing the risks of sharing data, despite none reporting to have experienced a data breach themselves.

The participants also used the availability heuristic when referencing personal and peers’ experiences with and outside of cancer. Some of these experiences led to negative perceptions of sharing, in particular, those who had had or heard of “bad experiences with insurance people in the past” [P05] that made them fearful of health or employment insurance denial. Other experiences led to positive reflections on data sharing. This was evident in some participants’ treatment issues that could have been mitigated if data had been shared, such as a life-threatening reaction or an adverse side effect. As Participant 13 noted, “that’s what’s important about data sharing, knowing who else out there has had that experience.” Another participant explained their willingness to share through their own treatment experience, recalling:

I’m one of the ones that treatment worked. And there is some mystification around it… good data is just so important.

P10

These experiences were salient to decision-making processes, given the way participants used these anecdotes as rationales for deciding whether to share their data.

The affect heuristic was identified primarily through voiced emotion around for-profit involvement in the data-sharing process. These feelings included the loss aversion our participants felt toward insurance company access, such as Participant 5’s previous experience with insurance “cast[ing] a negative light on the insurance industry” [P05], and Participant 13’s feeling that insurance access “immediately turns [them] off” [P13]. Other participants felt uneasy about the sale of data, with one participant expressing, “this whole selling and stuff, that kind of scares me” [P04], and another who felt that “tying finance to personal data it just doesn’t feel right.” [P10]. Another felt “spiteful” toward for-profit companies [P11], wanting to limit their access to data, and another described for-profit activity as “sinister” [P19]. Many participants used their affective pool of feelings to explain their intentions. We found that the availability and affect heuristics triggered a more negative intention when the participants felt a distrust, privacy concern, or loss aversion, and positively when the participants found a personal benefit or collective benefit to sharing data.

## Discussion

### Implications of Findings for Consent

We identified qualitative patterns in the heuristics that patients with cancer use for health data sharing and how they impact decisions to share. Data-sharing themes found in our transcripts are not new to the data-sharing literature: research has found that individuals are motivated by altruism, are hesitant to share with for-profit entities, and want greater control over the process [31]. What makes our findings novel is the consideration of how heuristics play a role in the generation of these preferences. By studying underlying intentions, this study shows how the mechanism used to construct seemingly rational preferences for data sharing is influenced by heuristic processing. For example, previously studied preferences for greater transparency into the use of health data may, according to this study, be grounded in heuristic responses despite the appearance of deliberation. The heuristics analyzed in these focus groups therefore shed new light on how preferences are formed and prioritized when deciding to share data.

Heuristics, or “decision shortcuts,” ultimately prove relevant in response to the complex and uncertain decision of sharing data. If participants are in fact using heuristics, then their decisions may not represent informed consent in the sense that they are not rationally deliberating on all outcomes to come to a utility-maximizing decision [49]. Instead of relying on what may be an impossible deliberation for patients, decision mechanisms can directly address these patterns of heuristic behavior by applying behavioral economic strategies, such as providing simplifying and salient information on the true risks and benefits at the time of consent. This approach can disrupt cognitive bias without asking individuals to deliberate all probabilities for a rational decision, as previous research has shown [10,23,40]. By respecting the boundedness of patients’ processing capabilities, this kind of consent process can reach meaningful consent that is not necessarily a rational process but produces rational outcomes that maximize the patient’s utility. As argued by Noah [49] (2016), it is important to recognize the obstacles to achieving perfect utility-maximizing consent and to instead encourage support for the “right” decision depending on the patient and the circumstances. The evidence found in this study can guide the testing of relevant behavioral economics strategies to manage the gap between the difficulty and yet necessity of giving deliberative informed consent.

### Limitations

These results are subject to limitations. First, the majority of our sample self-reported as white and of higher education and income, and participants skewed toward older age. Our findings lack perspectives that may be systematically different from what we captured [50,51]. Second, we did not put a limit on the length of time since the patients’ diagnosis or last treatment, which may have resulted in recall bias. Third, we chose to sample from patients with cancer and cancer survivors, who have unique experiences with their health and research in a way that may not be generalizable to individuals outside of this population [8,52,53]. Fourth, these findings are dependent on the subjectivity of the researchers’ applications of the heuristic framework. It is possible that the researchers confounded some of the heuristics, due to their similarities and the definitional nuances the researchers chose to apply. We attempted to mitigate this by having two researchers conduct independent analyses, applying a set of definitions informed by the literature, and comparing these two analyses during norming sessions. Finally, owing to study timelines and feasibility, we conducted all focus groups in English language.

Although not a limitation, we experienced some challenges with the use of Zoom for focus groups. This included the delayed and disruptive identification of one participant and another participant with poor sound quality. Following this first group, we developed a new identification process and were clearer about our expectations for sound quality from our participants, which resulted in smoother discussions in the next two groups.

### Conclusion

This study investigated whether and which heuristic processes are used in intentions to share health data for research. By applying a novel heuristic perspective, this research illuminates the influence of heuristic processes in the formation of preferences around data sharing. Future research can expand this work beyond intentions to share, observing how these heuristics may or may not be evidenced in actual decision-making behavior. Our findings also have implications for future practice, where the design of consent mechanisms for sharing health data should consider the role of heuristic processes in this multifactorial decision, and how behavioral economics strategies can be used to encourage more meaningful patient engagement with their decision-making.

## Appendix

Multimedia Appendix 1 Focus group consent form.

Multimedia Appendix 2 Demographic screening survey.

Multimedia Appendix 3 Semistructured focus group topic guide.

## Abbreviations

CLEO: the Canadian Network for Learning Healthcare Systems and Cost Effective ‘Omics Innovation
COREQ: Consolidated Criteria for Reporting Qualitative Research
REDCap: Research Electronic Data Capture

## Data Availability

Data sharing is not applicable to this article as no datasets were generated or analyzed during this study.

## References

[1] Shilo S, Rossman H, Segal E (2020). Axes of a revolution: challenges and promises of big data in healthcare. Nat Med.

[2] El-Galaly TC, Cheah CY, Villa D (2019). Real world data as a key element in precision medicine for lymphoid malignancies: potentials and pitfalls. Br J Haematol.

[3] Daniels H, Jones KH, Heys S, Ford DV (2021). Exploring the use of genomic and routinely collected data: narrative literature review and interview study. J Med Internet Res.

[4] Hulsen T, Jamuar S, Moody A, Karnes JH, Varga O, Hedensted S, Spreafico R, Hafler DA, McKinney EF (2019). From big data to precision medicine. Front Med (Lausanne).

[5] Deverka PA, Majumder MA, Villanueva AG, Anderson M, Bakker AC, Bardill J, Boerwinkle E, Bubela T, Evans BJ, Garrison NA, Gibbs RA, Gentleman R, Glazer D, Goldstein MM, Greely H, Harris C, Knoppers BM, Koenig BA, Kohane IS, La Rosa S, Mattison J, O'Donnell CJ, Rai AK, Rehm HL, Rodriguez LL, Shelton R, Simoncelli T, Terry SF, Watson MS, Wilbanks J, Cook-Deegan R, McGuire AL (2017). Creating a data resource: what will it take to build a medical information commons?. Genome Med.

[6] Rosenbaum S (2010). Data governance and stewardship: designing data stewardship entities and advancing data access. Health Serv Res.

[7] Angst CM, Agarwal R (2009). Adoption of electronic health records in the presence of privacy concerns: the elaboration likelihood model and individual persuasion. MIS Q.

[8] Anderson CL, Agarwal R (2011). The digitization of healthcare: boundary risks, emotion, and consumer willingness to disclose personal health information. Inf Syst Res.

[9] McGraw D, Mandl KD (2021). Privacy protections to encourage use of health-relevant digital data in a learning health system. NPJ Digit Med.

[10] Kehr F, Kowatsch T, Wentzel D, Fleisch E (2015). Blissfully ignorant: the effects of general privacy concerns, general institutional trust, and affect in the privacy calculus. Info Systems J.

[11] Dinev T, McConnell AR, Smith HJ (2015). Informing privacy research through information systems, psychology, and behavioral economics: thinking outside the APCO box. Inf Syst Res.

[12] von NJ, Morganstern O (1953). Theory of Games and Economic Behavior.

[13] Dinev T, Hart P (2006). An extended privacy calculus model for e-Commerce transactions. Inf Syst Res.

[14] Simon HA (1979). Rational decision making in business organizations. Am Econ Rev.

[15] Tversky A, Kahneman D (1974). Judgment under uncertainty: heuristics and biases. Science.

[16] Simon HA (1957). Models of Man.

[17] Kahneman D, Tversky A (1979). Prospect Theory: an analysis of decision under risk. Econometrica.

[18] Kahneman D (2011). Thinking, Fast and Slow.

[19] Simon H (1990). Invariants of human behavior. Annu Rev Psychol.

[20] Kahneman D (2003). A perspective on judgment and choice: mapping bounded rationality. Am Psychol.

[21] Smith HJ, Dinev T, Xu H (2011). Information privacy research: an interdisciplinary review. MIS Q.

[22] Fishbein M, Ajzen I, Ajzen I, Fishbein M (1980). Predicting and understanding consumer behavior: attitude-behavior correspondence. Understanding Attitudes and Predicting Social Behavior.

[23] Acquisti A, John LK, Loewenstein G (2013). What is privacy worth?. J Legal Stud.

[24] Gambino A, Kim J, Sundar SS, Ge J, Rosson M (2016). User disbelief in privacy paradox: heuristics that determine disclosure.

[25] Sundar SS, Kim J, Rosson MB, Molina MD (2020). Online privacy heuristics that predict information disclosure.

[26] Adjerid I, Peer E, Acquisti A (2018). Beyond the privacy paradox: objective versus relative risk in privacy decision making. MIS Q.

[27] Canadian Network for Learning Healthcare Systems and Cost-Effective 'Omics Innovation (CLEO).

[28] Marmion V, Bishop F, Millard DE, Stevenage SV (2017). The cognitive heuristics behind disclosure decisions. In Social Informatics. SocInfo.

[29] Metzger MJ, Flanagin AJ, Medders R (2010). Social and heuristic approaches to credibility evaluation online. J Commun.

[30] Patton MQ (2015). Qualitative Research and Evaluation Methods.

[31] Hermansen A, Regier DA, Pollard S (2022). Developing data sharing models for health research with real-world data: a scoping review of patient and public preferences. J Med Syst.

[32] Glaser BG (1965). The constant comparative method of qualitative analysis. Soc Probl.

[33] Clarke V, Braun V (2017). Thematic analysis. J Posit Psychol.

[34] Braun V, Clarke V (2006). Using thematic analysis in psychology. Qual Res Psychol.

[35] Braun V, Clarke V (2022). Toward good practice in thematic analysis: avoiding common problems and be(com)ing a knowing researcher. International Journal of Transgender Health.

[36] Lincoln YS, Guba EG (1985). Naturalistic Inquiry.

[37] Tong A, Sainsbury P, Craig J (2007). Consolidated criteria for reporting qualitative research (COREQ): a 32-item checklist for interviews and focus groups. Int J Qual Health Care.

[38] (2018). NVivo Qualitative Data Analysis Software.

[39] Saunders B, Sim J, Kingston T (2018). Saturation in qualitative researchxploring its conceptualization and operationalization. Qual Quant.

[40] Finucane ML, Alhakami A, Slovic P, Johnson SM (2000). The affect heuristic in judgments of risks and benefits. J Behav Decis Making.

[41] Acquisti A, John LK, Loewenstein G (2012). The impact of relative standards on the propensity to disclose. J Mark Res.

[42] Gigerenzer G, Todd PM, Gigerenzer G, Todd PM, The ABC Research Group (1999). Fast and frugal heuristics: the adaptive toolbox. Simple Heuristics That Make Us Smart.

[43] Joeckel S, Dogruel L, Bowman ND (2016). The reliance on recognition and majority vote heuristics over privacy concerns when selecting smartphone apps among German and US consumers. Inf Commun Soc.

[44] Kahneman D, Tversky A (1972). Subjective probability: a judgment of representativeness. Cogn Psychol.

[45] Dogruel L, Joeckel S, Vitak J (2017). The valuation of privacy premium features for smartphone apps: the influence of defaults and expert recommendations. Comput Hum Behav.

[46] Sundar SS, Metzger MJ, Flanagin AJ (2007). The MAIN Model: a heuristic approach to understanding technology effects on credibility. Digital Media, Youth, and Credibility.

[47] Brandimarte L, Acquisti A, Loewenstein G, Babcock L (2009). Privacy concerns and information disclosure: an illusion of control hypothesis. Dissertation. Carnegie Melon University.

[48] Wang L, Hu HH, Yan J, Mei MQ (2019). Privacy calculus or heuristic cues? The dual process of privacy decision making on Chinese social media. J Enterp Inf.

[49] Noah BA (2016). The (ir)rationality of (un)informed consent. Quinnipiac Law Review.

[50] Hudson M, Garrison N, Sterling R, Caron NR, Fox K, Yracheta J, Anderson J, Wilcox P, Arbour L, Brown A, Taualii M, Kukutai T, Haring R, Te Aika B, Baynam GS, Dearden PK, Chagné D, Malhi RS, Garba I, Tiffin N, Bolnick D, Stott M, Rolleston AK, Ballantyne LL, Lovett R, David-Chavez D, Martinez A, Sporle A, Walter M, Reading J, Carroll SR (2020). Rights, interests and expectations: indigenous perspectives on unrestricted access to genomic data. Nat Rev Genet.

[51] Priniski JH, Holyoak KJ (2022). A darkening spring: how preexisting distrust shaped COVID-19 skepticism. PLoS One.

[52] Hay AE, Leung YW, Pater JL, Brown MC, Bell E, Howell D, Kassam Z, Willing S, Tian C, Liu G (2017). Linkage of clinical trial and administrative data: a survey of cancer patient preferences. Curr Oncol.

[53] Köngeter A, Schickhardt C, Jungkunz M, Bergbold S, Mehlis K, Winkler EC (2022). Patients' willingness to provide their clinical data for research purposes and acceptance of different consent models: findings from a representative survey of patients with cancer. J Med Internet Res.

[54] Covidence Systematic Review Software, Veritas Health Innovation, Melbourne, Australia.

[55] Haddaway N, Grainger M, Gray C (2021). citationchaser: An R package and Shiny app for forward and backward citations chasing in academic searching. Zenodo.

