# Supplementary material for: Heuristics Identified in Health Data–Sharing Preferences of Patients With Cancer: Qualitative Focus Group Study
Source: J Med Internet Res. 2024 Dec 17;26:e63155. doi: 10.2196/63155 (PMC11688599; doi:10.2196/63155)
Supplement: Multimedia Appendix 1 [file jmir_v26i1e63155_app1.pdf]

Canadian Network for Learning Healthcare Systems and Cost-Effective 'Omics Innovation

Participant Information and Consent Form

Preferences for Secured Data Sharing Platforms and Patient Control of Data in Precision Oncology

Principal Investigator: **Dean Regier,** PhD
Associate Professor, School of Population and Public Health, UBC
Scientist, Cancer Control Research, BC Cancer Research Centre
Contact number: 604-675 8000 ext. 7079
Email: dregier@bccrc.ca

Co-Investigator: **Samantha Pollard,** PhD
Cancer Control Research, BC Cancer
Contact number: 604-675 8000 ext. 7050
Email: spollard@bccrc.ca

Dear prospective participant,

Researchers at BC Cancer and the University of British Columbia (UBC) are asking for your help with a research study. We want to find out what people think about sharing their personal data to support precision oncology. This study is a part of the Canadian Network for Learning Healthcare Systems and Cost-Effective 'Omics Innovation (CLEO) and is funded by Genome British Columbia / Genome Canada [G05CHS].

Precision oncology uses genetic and personal health data to individualize cancer prevention and treatment. Compared to traditional prevention and treatment selection that ignores genetic information, precision oncology may help patients live longer or have better quality of life. Our ability to use precision oncology depends on our understanding of how genetics influences cancer risk, prognosis and response to treatment. Secure sharing of personal health data can allow researchers to build this knowledge base for precision oncology.

The goal of this research is to understand what is important to people when thinking about their data being stored, shared and accessed to support precision oncology research.

What would my participation involve?

We are inviting you to participate in one focus group. The focus group will be conducted virtually, using UBC Zoom. During the focus group, you will be asked to discuss your opinions about the use of data-sharing platforms for precision oncology. You do not need to know anything about data-sharing platforms or technologies, or precision oncology, and do not need

to prepare in advance. Before the focus group, we may send you some brief background materials to review.

The focus group will last approximately 90 minutes.

We will also ask you to complete a brief questionnaire asking about yourself. If there are any questions that you would prefer not to answer, you can leave them blank. You do not need to provide any information you are not comfortable with.

After the focus group has been conducted, we may contact you again to ask if you would be interested in providing feedback about the focus group findings. A member of the research team will send you a summary of the results and will ask for feedback. Even if you participate in the focus group, you are not required to provide feedback, afterwards.

Why is my participation important?

Your participation will help us understand what is important to people when thinking about their personal data being stored and shared among researchers and doctors. This information will tell technology developers and decision-makers, such as health authorities and governments, what factors that need to be considered when designing new data sharing technologies and incorporating them into our healthcare system.

Are there any possible harms or discomforts in this study?

Participation in this study is completely voluntary. You are not required to participate and, if you do, you may withdraw from the study at any time without having to give a reason.

Participation in this study does not affect your medical care. You will continue to receive the best care your doctor and medical team can provide, regardless of whether or not you participate.

If you decide to participate, you do not have to talk about anything that you do not feel comfortable with.

Compensation

You will receive a one-time $100 honorarium for your participation.

How will you protect my confidentiality?

The focus group will be audio recorded and professionally transcribed. Before analyzing these recordings, the research team will remove your name and other personal information. While we

encourage participants not to share the content of the discussion to people outside of the focus group, we cannot control what other participants do with the information discussed.

All files will be transferred and stored to protect your confidentiality as according to the information technology service policies of the Provincial Health Services Authority. Recordings and notes taken during the focus group will be stored electronically on a password protected server by members of the research team. Audio files and other data will be stored on a secure server by the research team for seven years after the completion of the study, after which point it will be destroyed. As part of our consent process, we will store your name, contact information, and a copy of the signed consent form. Once you've completed this consent form, a your electronic consent form will be stored in secured networks in the Provincial Health Services Authority in Vancouver, BC. Only authorized personnel will be able to access it.

Your rights to privacy are protected by federal and provincial laws which require protections to ensure your privacy is respected. These laws also give you the right of access to the information that has been collected. Further details about these laws are available on request to the Principal Investigator.

We are asking to collect your email address. We will communicate with you via email about this study, and we will send you the electronic consent form and Zoom meeting invitation if you decided to participate in the study.

Although you may not be aware of this fact, emails sent to some webmail services (e.g. Gmail, Hotmail, etc.), may be stored/routed outside of Canada (for example, in the United States). Due to the fact that future emails will contain personal information about you, including your name, the Freedom of Information and Protection of Privacy Act requires that we obtain your consent. We will only send your personal information to the email address you have provided to us. All of the information which you provide to us will be kept completely confidential. Providing your email address means that you voluntarily agree and give your consent for the study team to email your personal information to you.

What does consent mean?

Your consent means that you understand what participation in this study will involve and agree to participate. You have the right to refuse to participate at any point during the study, including up to two weeks after the focus group session. If you withdraw your consent after the focus group, your information will not be used in the analysis and your comments will be removed from the transcripts of the focus group conversations.

Who can I contact for more information?

The project contact is:	The project's principal investigator is:
Samantha Pollard<br>Co-Investigator<br>BC Cancer<br>Phone: 604-675-8000 ext. 7050<br>Email: spollard@bccrc.ca	Dean Regier, PhD<br>Principal Investigator<br>BC Cancer, and UBC<br>Phone: 604-675-8000 ext. 7079<br>Email: dregier@bccrc.ca

You may contact either of the above if you desire additional information about your participation in the project.

For privacy related questions or questions about your rights as a research participant, you can contact the BC Cancer Research Ethics Board (REB) at reb@bccancer.bc.ca, or 604-877-6284. Please reference the study number H20-00861 when contacting the REB so the staff can better assist you.

Participant Consent

I have read and understood the subject information and consent form. I have had sufficient time to consider the information provided and to ask for advice if necessary. I have had the opportunity to ask questions and have had satisfactory responses to my questions. I understand that all of the information collected will be kept confidential and that the results will only be used for research objectives. I understand that my participation in this study is voluntary and that I am completely free to refuse to participate or to withdraw from this study at any time without changing in any way the quality of care that I receive. I understand that I may ask questions about this study in the future. I understand that I am not waiving any of my legal rights as a result of signing this consent form.

I will receive a signed copy of this consent form including all attachments, for my own records.

I consent to participate in the focus group.

______________________	____________________		__________
Participant's Signature	Printed name		Date
______________________	____________________	____________	__________
Signature of<br>Person Obtaining Consent	Printed name	Study Role	Date

Consent for re-contact for future research

The research team plans to conduct future studies in related topics. There may be opportunities for you to participate in future research, if you are interested. We would like to ask if you are willing to be contacted about future research studies. If you agree, we will retain your contact information. Your information will be securely stored on a password protected computer and will only be available to the study team. You may change your mind at any time and request your contact information be removed from our records. Your decision to provide your contact information for the purposes of future research participation has no bearing on your participation in the study, today.

Name:___________________ Date:______________________

☐ I <u>do not</u> wish to be contacted about future studies

☐ I agree to be contacted about future research studies

I prefer to be contacted by: ☐Telephone ☐Email

Email:_____________________________

Telephone:________________________

Best time to be contacted (circle all that apply): Morning Afternoon Evening Weekend

Signature:______________________________
