# Supplementary material for: Heuristics Identified in Health Data–Sharing Preferences of Patients With Cancer: Qualitative Focus Group Study
Source: J Med Internet Res. 2024 Dec 17;26:e63155. doi: 10.2196/63155 (PMC11688599; doi:10.2196/63155)
Supplement: Multimedia Appendix 2 [file jmir_v26i1e63155_app2.pdf]

Cleo Focus Group Screening Questions

Please complete the survey below.

Thank you!

CLEO Focus Group Participant Eligibility Screener

Please help us ensure this research captures a diversity of perspectives and values across Canada by responding to the questions below.

1) Full Name: ______________________________

2) Which Canadian province or territory do you currently live in?
- ◯ Alberta
- ◯ British Columbia
- ◯ Manitoba
- ◯ New Brunswick
- ◯ Newfoundland & Labrador
- ◯ Northwest Territories
- ◯ Nova Scotia
- ◯ Nunavut
- ◯ Ontario
- ◯ Prince Edward Island
- ◯ Quebec
- ◯ Saskatchewan
- ◯ Yukon

3) 2. What is your current age?
- ◯ Under 18 years
- ◯ 18 - 24
- ◯ 25 - 34
- ◯ 35 - 44
- ◯ 45 - 54
- ◯ 55 - 64
- ◯ 65 - 74
- ◯ 75 - 84
- ◯ 85 - 94
- ◯ 95 or older
- ◯ I prefer not to answer

4) 3. Please select the option that best describes your gender identity:
- ◯ Male
- ◯ Female
- ◯ Gender-fluid
- ◯ Nonbinary
- ◯ Trans man
- ◯ Trans woman
- ◯ Two-spirit
- ◯ Prefer to self-describe
- ◯ I prefer not to answer

5) If you selected 'Prefer to self-describe' for Question 3, please specify: ______________________________

6) 4. What type(s) or cancer have you been diagnosed with? ______________________________

7) 5. What racial or cultural group do you identify with (can select up to four)?

- ☐ Indigenous (First Nations, Métis, Inuk / Inuit)
- ☐ White (e.g. Scottish, Polish, French)
- ☐ Black (e.g. Haitian, Afro-Caribbean, Somali, Afro-Canadian)
- ☐ Latin American (e.g. Guatemalan, Brazilian, Colombian)
- ☐ South Asian (e.g. East Indian, Pakistani, Sri Lankan)
- ☐ East or South-East Asian (e.g. Chinese, Japanese, Malaysian, Vietnamese)
- ☐ Middle Eastern and West Asian (e.g. Iraqi, Iranian, Afghani)
- ☐ Other (please specify)
- ☐ Prefer not to answer

8) If you selected 'Other' for Question 5, please specify: ________________________

6. Please select all dates and times you are available to participate in this focus group.

9) a) Which times of the day work best for you?

- ☐ Mornings (9:00 AM to 12:00 PM)
- ☐ Early afternoons (12:00 PM to 3:00 PM)
- ☐ Late afternoons (3:00 PM to 6:00 PM)

10) b) Which days of the week generally work for you?

- ☐ Mondays
- ☐ Tuesdays
- ☐ Wednesdays
- ☐ Thursdays
- ☐ Fridays

11) c) If you would like to provide further details about your availability, please describe them below: ______________________________
