# Supplementary material for: Heuristics Identified in Health Data–Sharing Preferences of Patients With Cancer: Qualitative Focus Group Study
Source: J Med Internet Res. 2024 Dec 17;26:e63155. doi: 10.2196/63155 (PMC11688599; doi:10.2196/63155)
Supplement: Multimedia Appendix 3 [file jmir_v26i1e63155_app3.pdf]

	Focus Group Question<br><br>What is important to you when deciding whether to allow access to your personal data for research purposes?	Optional Prompt(s)
Introductions	☐ *Following research team introductions:* Please introduce yourself and let us know if there is anything you are particularly interested in talking about today<br><br>☐ Zoom poll: What are your first impressions of permitting access to your health data for research purposes? *(1 = very negative impression; 2 = negative impression; 3 = neutral; 4 = positive impression; 5 = very positive impression) After posting anonymous results*: Can you tell us why?	***Note taker to document topics to probe on during the discussion***<br><br>Does it matter who is requesting access? Does it matter what purpose they have for requesting access? ***probe why these specifics matter to the individual***
Expectations and concerns<br><br><br>Social norms heuristic	Motivation and willingness to allow access:<br>☐ Can you describe **what might motivate you** to allow access to your health data for research?<br><br>☐ Would your decision to allow access to your health data be influenced if you found out if most Canadians generally contribute their data to research projects?<br><br>☐ Can you describe **what might prevent you** from allowing researchers to access your information?<br><br>Expectations for allowing access:<br>☐ **What kinds of benefits,** if any, would you expect? (e.g. personal benefits? Community benefits? access to treatment, financial incentives, alternative forms of reimbursement?)<br><br>Decision-making:<br><br>☐ **What information would you want in advance** of being able to decide about allowing your data to be used for research?	What types of data would you be willing to allow access to, if any?<br><br>***probe for personal v. community v. future population benefit, including specific aspects of personal benefit (e.g. QOL)***<br><br>What if you found out that one of your friends with cancer permitted access to their data? Why?<br><br>What, if anything, could be done to mitigate barriers or alleviate your concerns?

Consent, control and incentivization	***Walk through graphic from education deck depicting how users may be able to control the use of data using the platform***<br><br>**Under which conditions, if any,** would you consider allowing:<br>☐ your data to be used without your consent?<br>☐ for-profit vs non-profit institutions to access your data? *(Is there anything in particular about for-profit access that concerns you? Why or why not?)*<br>☐ access to your data on a more case-by-case basis?<br><br>☐ Would your willingness to allow access to your data change if you were offered incentives? If so, how?	Do you envision any drawbacks to an approach that allows you to control each use of your data?<br><br>Are there specific safeguards that could be put in place that would make you feel comfortable about allowing your data to be used without your consent?<br><br>E.g. Universities, Government institutions like BC Cancer, pharmaceutical companies?<br>***probe participants to raise the kinds of incentives they may be interested in receiving***<br>☐ What kind of compensation or incentives might you be interested in? (health? Financial? Help for future patients? External but non-financial?)
Design of data sharing platform	***Refer to blockchain slide***<br>☐ What features of a data sharing platform would be important to you?	What features are not important to you? Why? **What would make a platform user-friendly**, in your opinion?
Affect and availability heuristic	☐ Taylor, a resident of BC, reads about a recent cyberattack on the Newfoundland health data system. The attack disrupted many services	***probe about LifeLabs breach / Newfoundland cyberattack*** How are

	across the province and the attackers stole patient data including birthdays, email addresses, and appointment information. Taylor is then notified that an Ontario researcher has contacted BC Cancer to access Taylor's genetic test data that may help the development of a new therapy for a cancer that runs in their family.<br>If you were Taylor, would you permit access to your data?	these attacks relevant to your decision?
Hot state (affect heuristic)	☐ Zoom poll: After this discussion, what are your impressions of permitting access to your health data for research purposes? *(1 = very negative impression; 2 = negative impression; 3 = neutral; 4 = positive impression; 5 = very positive impression)*<br>*After posting anonymous results:* Can you tell us why?	
Closing	Is there anything else that you would like to talk about, that we have not discussed here today?	
